# Aqueous Phase Synthesis and Enhanced Field Emission Properties of ZnO-Sulfide Heterojunction Nanowires

**DOI:** 10.1038/srep29470

**Published:** 2016-07-08

**Authors:** Guojing Wang, Zhengcao Li, Mingyang Li, Chienhua Chen, Shasha Lv, Jiecui Liao

**Affiliations:** 1 State Key Lab of New Ceramic and Fine Processing, School of Materials Science & Engineering, Tsinghua University, Beijing 100084, China; 2 Key Lab of Advanced Materials (MOE), School of Materials Science and Engineering, Tsinghua University, Beijing 100084, China; 3 Department of Engineering Physics, Tsinghua University, Beijing 100084, China

## Abstract

ZnO-CdS, ZnO-ZnS, and ZnO-Ag_2_S core-shell heterojunction structures were fabricated using low-temperature, facile and simple aqueous solution approaches. The polycrystalline sulfide shells effectively enhance the field emission (FE) properties of ZnO nanowires arrays (NWAs). This results from the formation of the staggered gap heterointerface (ZnO-sulfide) which could lead to an energy well at the interfaces. Hence, electrons can be collected when an electric field is applied. It is observed that ZnO-ZnS NWAs have the lowest turn-on field (3.0 Vμm^−1^), compared with ZnO-CdS NWAs (6.3 Vμm^−1^) and ZnO-Ag_2_S NWAs (5.0 Vμm^−1^). This may be associated with the pyramid-like ZnS shell which increases the number of emission nanotips. Moreover, the Fowler-Nordheim (F-N) plot displays a nonlinear relationship in the low and high electric field regions caused by the double well potential effect of the heterojunction structures.

The core-shell heterostructures have attracted much attention due to its consisting of two components with distinct functionalities^1^,^2^ that often exhibit enhanced characteristics, such as emission efficiency^3^ and high electron mobility^4^. These are the key factors for many device performances^5^,^6^,^7^ when compared to individual ones. Recently, wide variety of core-shell heterostructures, such as Si-NiSi^8^, Si-SiO_2_^9^, ZnO-graphite^10^, ZnO-SnO_2_^11^, ZnO-CdS-Ag_2_S^12^, ultrananocrystalline diamond needles-ZnO nanorod^13^, ZnO-Al_2_O_3_ core-shell nanowires (NWs)^14^, and ZnO-CdTe core-shell nanocable arrays^15^ have been investigated. Synthesizing such core-shell heterostructures is not only significant for scientific studies but also for a wide field of advanced functional devices, for example, solar cells, emitters, and so forth. Particularly, electron field emission (FE) emitters, utilizing suitable source materials, electrons are produced via FE by applying a high electrostatic field to lead electrons to tunnel from the cold cathode materials surface into vacuum, are closely linked with the surface morphology, structure and Fermi energy level of the materials.

In recent years, the high-performance cold cathode materials like carbon nanotubes and semiconductor nanowires or nanotubes, which possess a low turn-on voltage and a high emission current, are one dimensional (1D) nanostructures^16^,^17^,^18^,^19^. The results from 1D nanostructures, which show the high aspect ratio and small radius of curvature, exactly meet the requirements of good FE performance^18^,^19^,^20^,^21^,^22^. The 1D ZnO nanostructures have been extensively studied in the FE area, due to its inexpensive, nontoxic, and excellent physical and chemical properties^23^,^24^,^25^,^26^,^27^,^28^. However, there are still some remaining problems in utilizing ZnO as emitters material. Primarily, ZnO possesses a high turn-on field (*E*_to_) due to the high work function of ~5.38 eV^18^,^25^,^26^,^27^,^28^. The typical scheme, such as doping and modifying, have been widely used to reduce the turn-on field (*E*_to_) of 1D ZnO nanostructures. According to the recent research, the ZnO-sulfide heterojunction structures display an amazing FE performance^29^,^30^. The turn-on fields are as low as 0.02 and ~2 V/μm for ZnO-ZnS heterojunction nanocone arrays^29^ and CdS nanoparticles deposited on 3D self-assembled ZnO nanorods^30^, respectively. To the best of our knowledge, the origin of the amazing FE properties for these heterojunction structures are still an open issue and the studies to compare the difference between these different kinds of ZnO-sulfide heterojunction structures has rarely been reported. Moreover, some sulfides which possess promising application in EF have not been paid much attention to, such as Ag_2_S^31^, which has a narrow *E*_g_ of 1.1 eV and shows excellent photoelectric properties.

In this study, three kinds of ZnO-sulfide (ZnO-CdS, ZnO-ZnS, and ZnO-Ag_2_S) core-shell heterojunction nanowire arrays (NWAs) have been fabricated and their FE properties have been systematically investigated. The FE properties of ZnO NWAs have been effectively improved by modifying with sulfide shell. Particularly, ZnO-ZnS NWAs have the lowest turn-on field, compared with ZnO-CdS and ZnO-Ag_2_S NWAs. The morphologies, structures and energy band structures of these core-shell heterojunction NWAs have been characterized and their effects on FE properties have also been discussed.

## Results and Discussion

### Morphological observations and structure

**Figure 1:**
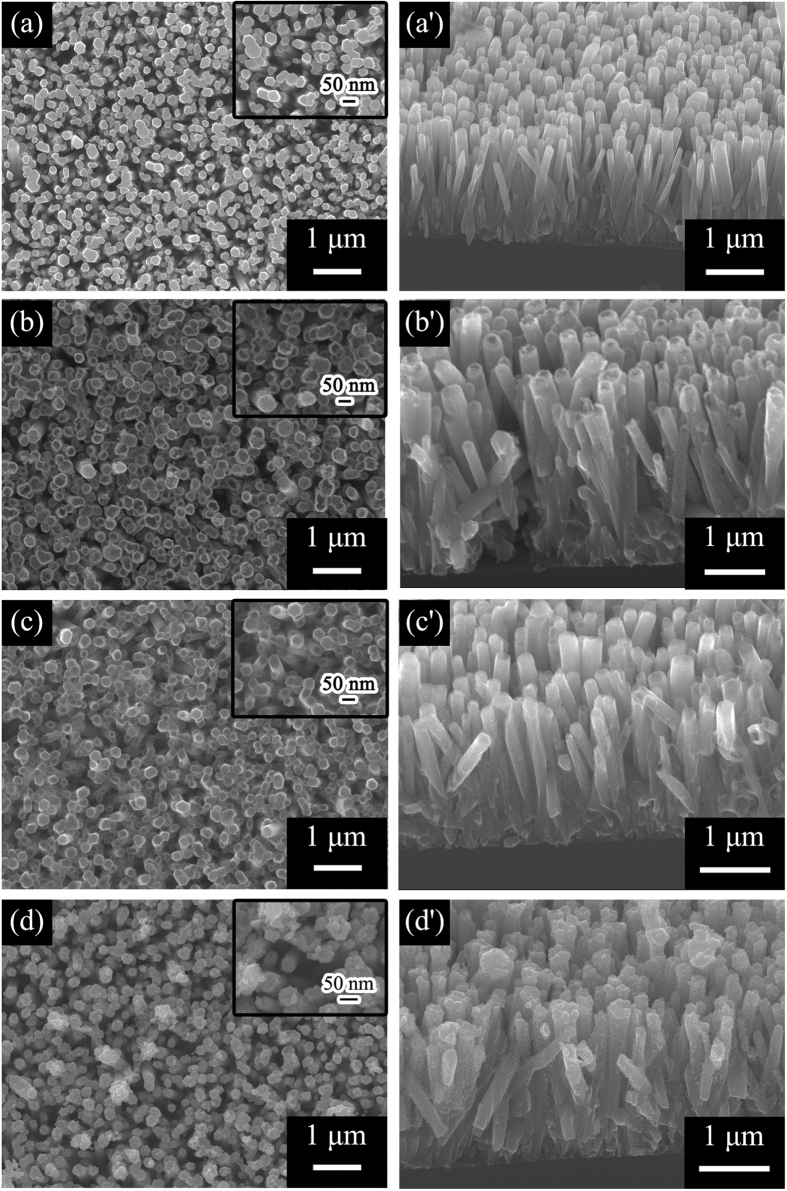
SEM images of ZnO, ZnO-CdS, ZnO-ZnS and ZnO-Ag_2_S NWAs for top section (**a**–**d**) and cross section (**a′**–**d′**). The insets of the (**a**–**d**) are the high magnification SEM image for top section.

The typical top and cross section microscopes electron microscopy (SEM) images of the as-prepared ZnO ((a) and (a’)), ZnO-CdS ((b) and (b’)), ZnO-ZnS ((c) and (c’)), and ZnO-Ag_2_S ((d) and (d’)) are shown in Fig. 1. The insets of Fig. 1a–d are high magnification SEM images. The length and the diameter of ZnO NWs are around 2.6 μm and 120 nm, respectively. CdS and ZnS formed a uniform shell layer on ZnO NWs, while Ag_2_S were uniformly deposited on ZnO NWAs as quantum dots (QDs). Some nanoparticles (NPs) are observed on the top of the ZnO-Ag_2_S NWAs due to the clustering of Ag_2_S QDs. The diameter of the NWs with sulfide shell is obviously increased.

**Figure 2:**
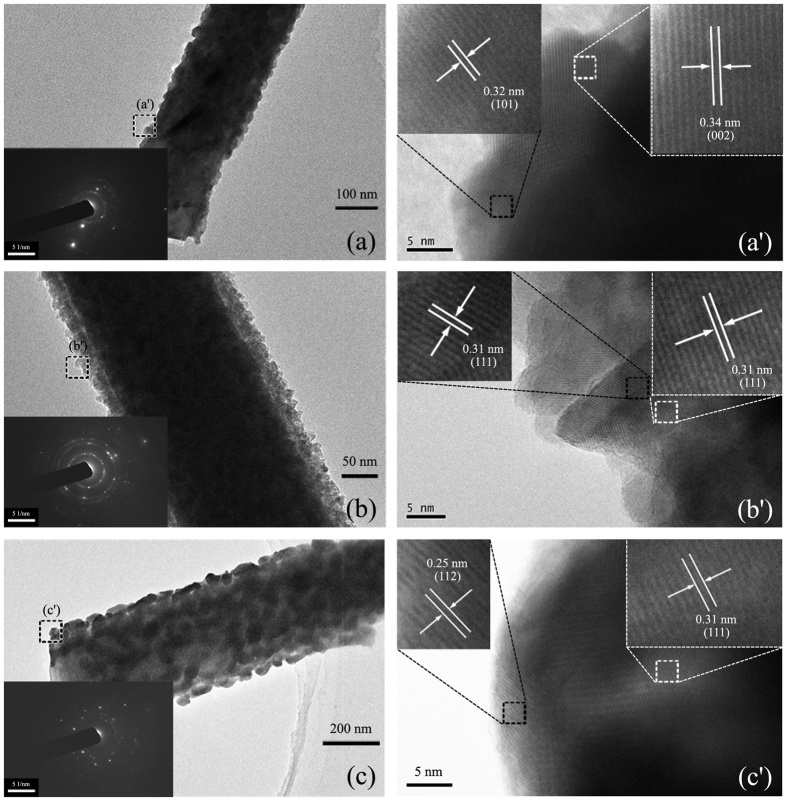
TEM characterization of ZnO-CdS, ZnO-ZnS and ZnO-Ag_2_S ((**a**–**c**): bright-field image, (**a′**–**c′**): high-resolution image from area marked in (**a**–**c**) for the sulfide shell. The insets of (**a**–**c**) are the selected area electron diffraction patterns. The insets of (**a′**–**c′**) are the high magnification TEM images.

To further observe the morphology and the surface, transmission electron microscopy (TEM) images of the ZnO-CdS ((a) and (a’)), the ZnO-ZnS ((b) and (b’)), and the ZnO-Ag_2_S ((c) and (c’)) are displayed in Fig. 2. As seen in the bright-field images as Fig. 2a–c, CdS and ZnS formed a highly uniform shell layer with a thickness around 30 nm on the ZnO NWs, whereas Ag_2_S formed large dots uniformly distributing on the surface of ZnO NWs with a mean diameter of ~30 nm. The ZnO-Ag_2_S NWs have the biggest diameter, because the Ag_2_S QDs are not monolayer. Further examination reveals that the surfaces of the three kinds of core-shell nanocomposites all have a relatively rough morphology, especially the ZnO-ZnS and the ZnO-Ag_2_S. The pyramid-like ZnS results in more nanotips on the surface. The insets of Fig. 2a–c are the selected area electron diffraction (SAED) pattern of the samples, which indicate that the shells of CdS, ZnS, and Ag_2_S QD are all polycrystalline. Two sets of zone diffraction patterns, corresponding to ZnO and CdS, have been observed in the SAED pattern of the ZnO-CdS resulting from the partial divorce of CdS from the ZnO NW during the preparation of the TEM sample. The high-resolution TEM images, exhibited in Fig. 2a′-c′, show the (101) and (002) planes of the hexagonal wurtzite CdS phase; the (111) face represents the cubic zinc blende ZnS and the (111) and (112) planes represent the monoclinic Ag_2_S phase, respectively (verified with reference data JCPDS 41-1049, 65-5476, and 14-0072). Due to the highly dense shells forming on the ZnO NW surface, the HRTEM images of ZnO NW could not be observed.

**Figure 3:**
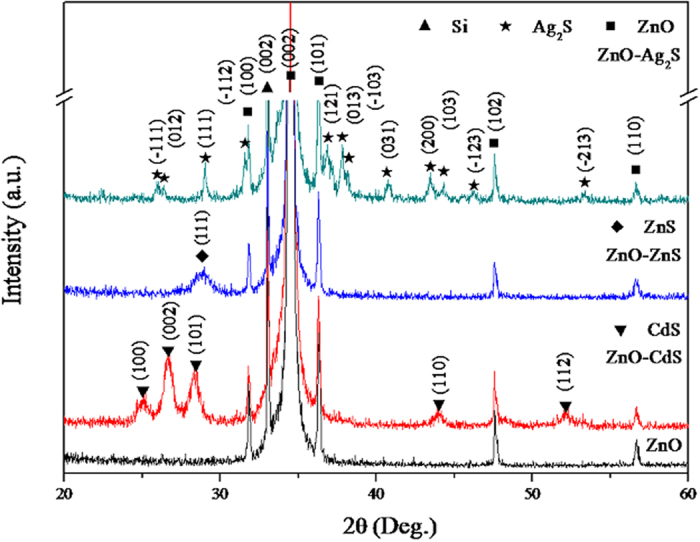
XRD patterns of ZnO, ZnO-CdS, ZnO-ZnS and ZnO-Ag_2_S NWAs.

In order to confirm the core-shell nanostructures, the X-ray diffraction (XRD) patterns of the as-prepared ZnO, ZnO-CdS, ZnO-ZnS, and ZnO-Ag_2_S NWAs are shown in Fig. 3. Apparently, there is no characteristic peak of any impurities. The peak locating at around 32.9° correspond to the (002) plane of Si(001) substrate. The ZnO diffraction peaks are clearly observed and can be indexed to hexagonal wurtzite ZnO (JCPDS 36-1451). The relatively high intensity of the peak corresponding to (002) planes of ZnO implies a preferred orientation of the crystallites. The pattern of the ZnO-CdS core-shell NWAs consists of two sets of diffraction peaks (ZnO and CdS) and the pattern of CdS diffraction peaks can be indexed to the hexagonal wurtzite structure (JCPDS 41-1049). The observed peaks can be assigned to the (100), (002), (101), (110), and (112) planes of the wurtzite phase CdS. The pattern of the ZnO-ZnS core-shell NWAs also consists of two sets of diffraction peaks (ZnO and ZnS) and the broadening peak at 28.6° matches well with the cubic zinc blende ZnS (JPCDS 65-5476). While, the peaks at 47.6° of (220) and 56.4° of (311) of ZnS crystal planes are overlapped with the diffraction peaks of (102) and (101) crystal planes of ZnO. The ZnO-Ag_2_S core-shell heterojunction is confirmed by the diffraction pattern and the diffraction peaks of Ag_2_S can be assigned to the monoclinic structure (JCPDS 14-0072) with (111), (112), (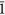
03), (200) and (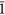
23) planes. From the XRD patterns of the ZnO-sulfide nanocomposites, all the Bragg peaks of ZnO of different samples located at the same degree. This indicates that the modification with sulfide shells does not affect the structure of crystalline ZnO NWAs.

**Figure 4:**
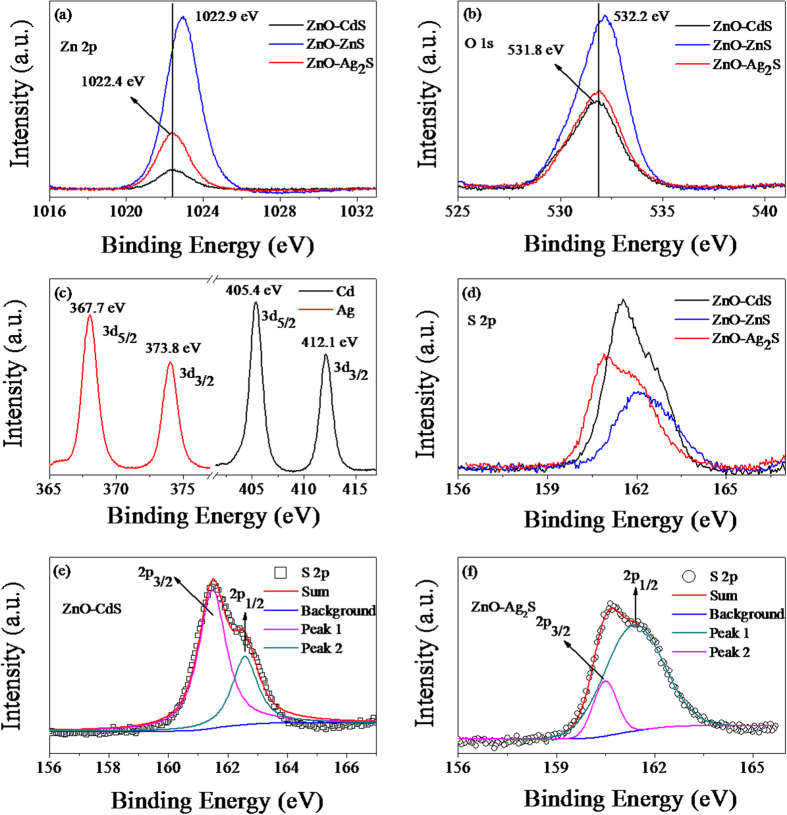
XPS spectra of ZnO-CdS, ZnO-ZnS and ZnO-Ag_2_S (**a**) Zn 2p; (**b**) O 1s; (**c**) Ag 3d and Cd 3d; (**d**) S 2p. (**e**,**f**) the fitting results of the element S for ZnO-CdS and ZnO-Ag_2_S, respectively.

To further clarify the elemental and chemical states of the composites, X-ray photoelectron spectroscopy (XPS) measurements were performed on the ZnO-CdS, ZnO-ZnS, and ZnO-Ag_2_S NWAs. In the survey scan, only C, Zn, O, S, and Cd (or Ag) elements were observed in the whole spectra and the corresponding high resolution spectra of Zn (a), O (b), Cd/Ag (c), and S (d) are plotted in Fig. 4. In the Zn 2p spectrum (Fig. 4a), a main peak is observed at a binding energy of 1022.1 eV for the ZnO-CdS and ZnO-Ag_2_S NWAs. However, the Zn 2p_3/2_ peak of the ZnO-ZnS moves to higher binding energy because of the ZnS shell. The peak in O 1s spectrum (Fig. 4b) of ZnO-ZnS weakly moves to higher binding energy (532.2 eV) compared with the ZnO-CdS and ZnO-Ag_2_S which both locate at 531.8 eV. This implies that the chemical states of ZnO core were weakly affected by the sulfide shells. For the ZnO-Ag_2_S, the Ag 3d spectrum contains two peaks, one at 367.7 eV for the Ag3d_5/2_ and the other at 373.8 eV for the Ag 3d_3/2_, which are in a good agreement with the published values of the Ag 3d signal in Ag_2_S compound^12^,^32^. The 3d_5/2_ and 3d_3/2_ spin-orbit splitting of the Cd 3d level occurs at the binding energies of 405.4 and 412.1 eV in the ZnO-CdS, respectively. The Cd 3d_5/2_ peak at the binding energy of 405.4 eV corresponds to Cd^2+^ combined with S^2−^33,34. Figure 4d shows the high resolution XPS spectra of S 2p peaks. Interestingly, the S 2p peaks of the ZnO-CdS and ZnO-Ag_2_S are asymmetric and can be divided into two peaks. The fitting results are given in Fig. 4e (ZnO-CdS) and f (ZnO-Ag_2_S). The centers of the S 2p peaks of the ZnO-CdS locate at 161.5 and 162.6 eV corresponding to the sulfur in CdS and ZnS, respectively. And the S 2p peaks of the ZnO-Ag_2_S centers at 160.5 and 161.4 eV corresponding to the sulfur in Ag_2_S and ZnS, respectively^12^,^32^,^35^. These results affirm that a trace amount of ZnS formed during the preparation process of the ZnO-CdS and ZnO-Ag_2_S.

### Field emission properties

**Figure 5:**
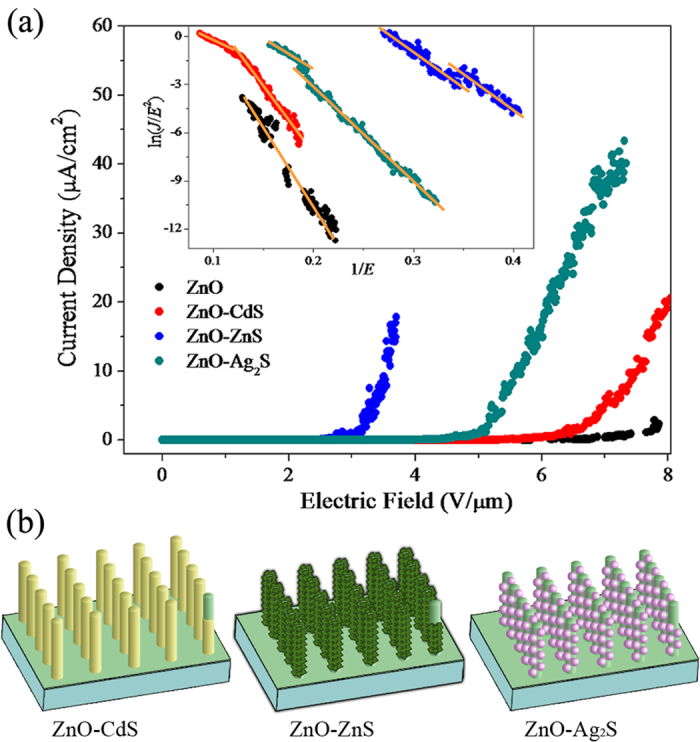
(**a**) The *J*-*E* behavior of ZnO, ZnO-CdS, ZnO-ZnS and ZnO-Ag_2_S NWAs. The inset of (**a**) is the F-N plots of ZnO, ZnO-CdS, ZnO-ZnS and ZnO-Ag_2_S NWAs. The solid lines are the fitting result. (**b**) The schematics of the structure of the ZnO-CdS, ZnO-ZnS and ZnO-Ag_2_S core-shell NWAs.

FE is that the electrons near the Fermi level emits to vacuum by quantum mechanical tunneling from a conducting/semiconducting emitter with the application of a very high external electric field (~10^5^ Vcm^−1^). During the FE process, the FE current-voltage characteristics were analyzed using the following equation^36^:


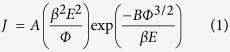


where *J* is the current density, *E* is the applied electric field, *A* = 1.56 × 10^−6^ (A eVV^−2^), *B* = 6.83 × 10^3^ (V μm^−1^eV^−2/3^), and *Φ* is the work function of the ZnO NWs, whose value is around 5.37 eV^18^,^25^,^26^,^27^,^28^. *β* is the field-enhancement factor, which is related to the emitter geometry, crystal structure, and spatial distribution of the emitting centers. Figure 5a presents the typical current density-electric field (*J*-*E*) characteristics of the as-prepared ZnO, ZnO-CdS, ZnO-ZnS, and ZnO-Ag_2_S. Negligible FE currents from as-prepared ZnO NWAs up to the maximum applied electric field were observed. The turn-on field (*E*_to_) is defined as the electric fields required to produce a current density of 1 μAcm^−2^. The *E*_to_ of the as-prepared ZnO, ZnO-CdS, ZnO-ZnS, and ZnO-Ag_2_S NWAs are shown in Table 1. It can be seen that the *E*_to_ of the NWAs is effectively reduced by the core-shell heterojunction structures. ZnO-ZnS NWAs have the lowest *E*_to_ (around 3.0 Vμm^−1^), followed by ZnO-Ag_2_S NWAs (5.0 Vμm^−1^), and ZnO-CdS NWAs (6.3 Vμm^−1^). Besides of the intrinsic properties of the sulfides, the turn-on field is found to be closely related with the structure and microtopography. Figure 5b gives the corresponding schematics of the surface microtopography of the ZnO-CdS, ZnO-ZnS, and ZnO-Ag_2_S. The pyramid-like ZnS shell can more widely increase the number of nanotips than the dot-like Ag_2_S and layer-like CdS, thus the turn-on field of the ZnO-ZnS is even lowered. This inference can be confirmed by comparing with similar published works, as provided in Table 1. Zhang *et al*.^29^ fabricated nanocone-like ZnO-ZnS arrays using chemical vapor deposition. Warule *et al*.^30^ synthesized the 3D nano-architectures ZnO modified with CdS nanoparticles via a facile single-step hydrothermal approach. Their samples possess different structures leading to slenderer and denser nanotips than our ZnO-sulfide NWAs. Thus, the *E*_to_ of their samples are smaller than our results. The vertical and uniform ZnO-sulfide core-shells NWAs in this work also possess small differences in microtopography and can improve the FE properties of ZnO NWAs to varied degrees. By optimizing the synthesis process, samples which possess slenderer and denser nanotips can be prepared to improve FE properties. For further confirmation and improvement of the FE properties, the same material with different surface microtopography and structure will be synthesized in our next work using different preparation methods.

The emission current-voltage characteristics were further analyzed by Fowler-Nordheim (F-N) equation which can be used to describe the linear relationship between ln(*J*/*E*^2^) and 1/*E*.


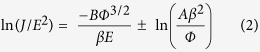


ln(*J*/*E*^2^) was plotted as a function of 1/*E*, as shown in the inset of Fig. 5a. It is notable that the F-N plot displays a nonlinear relation in the low and high electric field regions. A down bending F-N plot is often observed for nanocomposites as electric field increasing, and it has been widely discussed in the literature^37^. This phenomenon can be explained by the double well potential effect^31^. Except the barrier potential between the surface and the vacuum level, there also forms a barrier potential between the ZnO core and the sulfide shell. When two different sorts of nano-material are combined, the total field enhancement factor *β* is the combination of two individual nanostructures^37^, presented as:


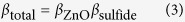


where *β*_ZnO_ and *β*_sulfide_ are the field enhancement factors of the ZnO core and the sulfide shell, respectively.

In many reports, only the linear region at high electric field is fitted with the F-N equation, while the region at low electric field is ignored. Thus, the *β* of the high electric field region (left part of plot) of the as-prepared ZnO, ZnO-CdS, ZnO-ZnS, and ZnO-Ag_2_S NWAs are calculated and shown in Table 1.

**Figure 6:**
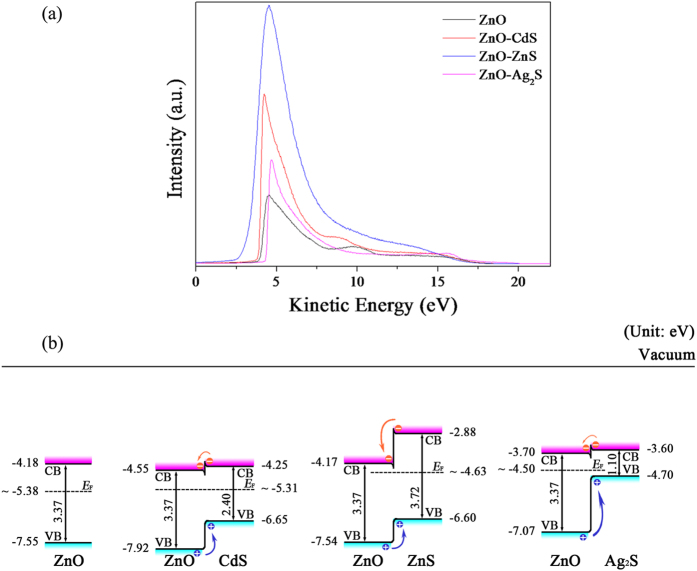
(**a**) A typical He (I) (*hv* = 21.22 eV) UPS spectra of ZnO, ZnO-CdS, ZnO-ZnS and ZnO-Ag_2_S NWAs taken with −10.0 V bias applied to the samples. The energy level diagrams of ZnO, ZnO-CdS, ZnO-ZnS and ZnO-Ag_2_S NWAs.

In order to reveal the carrier transfer path, the energy level diagrams of the samples were determined by ultraviolet photoelectron spectroscopy (UPS) measurement. Figure 6a illustrates the UPS spectra of the as-prepared ZnO, ZnO-CdS, ZnO-ZnS, and ZnO-Ag_2_S NWAs. Due to the strong surface charge effect, the work function of ZnO is measured to be around 4.11 eV which is lower than the reported value of ~5.38 eV^18^,^25^,^26^,^27^,^28^. Based on the highest occupied molecular orbitals (HOMOs), the maximum energy level of valence band (*E*_v_) of the as-prepared ZnO has been deduced to be 7.55 eV (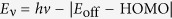). The minimum energy level of conduction band (*E*_c_) was calculated using the band gap (*E*_g_ = 3.37 eV)^18^,^25^,^26^,^27^,^28^. The maximum energy levels of valence band of the three kinds of ZnO-sulfide NWAs weakly shift from the *E*_v_ of the as-prepared ZnO. This is attributed to the effect of the heterojunction structure. The energy level diagrams of the samples are presented in Fig. 6b. The maximum energy levels of the valence band of CdS, ZnS, and Ag_2_S are taken as 6.65 eV^30^, 6.60 eV^29^ and 4.70 eV^31^, respectively. The band gaps of CdS, ZnS, and Ag_2_S are taken as 2.40 eV^30^, 3.72 eV^29^ and 1.10 eV^31^, respectively. As shown in Fig. 6b, a staggered gap heterointerface (ZnO-sulfide) formed, which could lead to a free barrier for hole transport and an energy well on the minimum energy level of conduction band that collects electrons when an electric field is applied. In addition, *E*_F_ weakly shifts to *E*_c_ when n and n type semiconductors form heterojunction structures. However, the energy band is bent at the interface, then *E*_F_ of the composite is leveled when n and p type semiconductors come into contact. This is the reason why ZnO-sulfide core-shell heterojunction structures effectively enhance the FE properties of ZnO NWAs.

## Conclusions

In this research, ZnO-CdS, ZnO-ZnS, and ZnO-Ag_2_S core-shell heterojunction NWAs were synthesized by aqueous solution approaches. These sulfide shells are polycrystalline and uniformly packed on the ZnO NWs. These sulfide shells induce a relatively rough surface and obviously increase the diameter of NWs, while do not affect the structure of ZnO NWs. The FE properties of ZnO NWAs have been effectively improved by modifying with sulfide shells. This is associated with it that the staggered gap heterointerface (ZnO-sulfide) which could lead to the energy well at the interfaces. Thus, electrons can be collected when an electric field is applied. ZnO-ZnS NWAs have the lowest *E*_to_ (around 3.0 Vμm^−1^), followed by ZnO-Ag_2_S NWAs (5.0 Vμm^−1^), and ZnO-CdS NWAs (6.3 Vμm^−1^). This may result from that the pyramid-like ZnS shell can widely increase the number of nanotips than the dot-like Ag_2_S and layer-like CdS. The F-N plot displays a nonlinear relation in the low and high electric field regions resulting from the double well potential effect of the heterojunction structures.

## Experimental

### Materials and Preparation

Synthesis of the ZnO-sulfide core-shell heterojunction NWAs takes two steps, the preparation of ZnO NWAs and the deposition of the sulfide. The former was carried out by a traditional solution approach, the setup of which was described in detail previously^18^. ZnO NWAs were prepared by firstly depositing a thin ZnO film on single crystal Si(100) substrates (thickness: 0.5 mm) at room temperature by radio frequency (RF) magnetron sputtering technology for 10 min. Then, by immersing the substrates with ZnO film into 200 ml 0.025 M aqueous solution of zinc nitrate [Zn(NO_3_)_2_•6H_2_O] and 0.025 M hexamethylenetetramine (C_6_H_12_N_4_) the hydrothermal process was conducted at 95 °C for 3 h. After the chemical reaction, the samples (ZnO NWAs) were rinsed with distilled water and dried.

### Synthesis of ZnO-CdS core-shell nanowire arrays

CdS was deposited by using aqueous solution of 0.02 M CdCl_2_ and 0.06 M NH_2_CSNH_2_. Firstly, the adequate NH_4_Cl aqueous solution was used as an agent to react with CdCl_2_ and form Cd(NH3)_4_^2+^. The Cd(NH_3_)_4_^2+^ can slowly release Cd^+^ and Cd^+^ covered ZnO nanowires homogenously. The aqueous was set at 70 °C and pH value was adjusted to 10 with NH_3_ aqueous solution. Secondly, NH_2_CSNH_2_ aqueous and as-grown ZnO NWs were added into the reaction system. Deposition was performed for 15 minutes with continuous stirring in a beaker. After deposition, the samples were washed with deionized water and dried with N_2_.

### Synthesis of ZnO-ZnS core-shell nanowire arrays

A simple two-step chemical solution reaction method was used to build ZnS-coated ZnO NWs with a self-assembling method. First, the as-prepared ZnO NWAs were immersed in 0.16 M sodium sulfide (Na_2_S) solution at 60 °C with magnetic stirring for 3 h. Then, the product was washed with deionized water. The second step was performed by immersing the above product into zinc nitrate (Zn(NO_3_)_2_) solution whose concentration was the same as the Na_2_S solution at 60 °C for 3 h. Lastly, the samples were dried at 40 °C in air.

### Synthesis of ZnO-Ag_2_S core-shell nanowire arrays

Ag_2_S was deposited on ZnO NWs using the successive ionic layer adsorption and reaction (SILAR) method at room temperature^38^. The ZnO NWAs were first immersed into 0.02 M Na_2_S, the anionic precursor solution, for 30 s so S^2−^ ions were absorbed on the ZnO NWs. Then ZnO NWAs were rinsed with deionized water. Next, the ZnO NWAs were immersed into 0.02 M AgNO_3_, the cationic precursor solution, for the same time. Ag^+^ ions reacted with adsorbed S^2−^ ions on ZnO NWs and formed Ag_2_S. Lastly, the ZnO-Ag_2_S NWAs were rinsed with deionized water. These four-steps are considered as one SILAR cycle and the cycle was repeated for 20 cycles.

### Characterization

The morphology and structure of the samples were characterized by field-emission microscopes electron microscopy (FE-SEM, JEOL-JSM 7001F), high-resolution transmission electron microscopy (HRTEM, JEM-2010, JEOL) and X-ray diffraction (XRD; SmartLab, Rigaku). The elemental and chemical states of the samples were evaluated by X-ray photoelectron spectroscopy (XPS, ESCALAB250Xi, Thermofisher Scientific). The energy levels were evaluated using ultraviolet photoelectron spectroscopy (UPS; ESCALab 250Xiusing, Thermo Scientific). A gas discharge lamp was used for UPS, with helium gas admitted and the He (I) (*hv* = 21.22 eV) emission line employed. The helium pressure in the analysis chamber during analysis was about 2 × 10^−8^ mbar. The data were acquired with −10.0 V bias.

### The Field Emission Properties Measurements

The field emission properties of the samples were measured in a vacuum chamber with base pressures below 5 × 10^−5^ Pa. The transparent conductive material (indium tin-oxide) serves as the anode electrode in the vacuum system. The distance between the sample and the anode electrode is ~200 μm.

## Additional Information

**How to cite this article**: Wang, G. *et al*. Aqueous Phase Synthesis and Enhanced Field Emission Properties of ZnO-Sulfide Heterojunction Nanowires. *Sci. Rep.* **6**, 29470; doi: 10.1038/srep29470 (2016).

## Figures and Tables

**Table 1:** Comparison of key parameters of our samples with similar studies.

Materials	Morphology	Turn-on Field Vμm^−1^	Enhancement Factor
ZnO^25^	NWAs	19.0	
ZnO^26^	NWAs	12.0 (0.1 μAcm^−2^)	
ZnO^27^	Needle like NWAs	18.0 (0.01 μAcm^−2^)	372
ZnO-ZnS^29^	Nanocone arrays	0.02 (10 μAcm^−2^)	5.6 × 10^4^
ZnO-CdS^30^	3D nano-architectures	2.0 (10 μAcm^−2^)	
ZnO*	NWAs	7.70 (1 μAcm^−2^)	850
ZnO-CdS*	NWAs	6.3 (1 μAcm^−2^)	2985
ZnO-ZnS*	NWAs	3.0 (1 μAcm^−2^)	1872
ZnO-Ag_2_S*	NWAs	5.0 (1 μAcm^−2^)	2244

^*^In this work.
